# Avoiding unscheduled transcription in shared promoters: *Saccharomyces cerevisiae* Sum1p represses the divergent gene pair *SPS18*-*SPS19* through a midsporulation element (MSE)

**DOI:** 10.1111/j.1567-1364.2009.00527.x

**Published:** 2009-07-03

**Authors:** Aner Gurvitz, Fumi Suomi, Hanspeter Rottensteiner, J Kalervo Hiltunen, Ian W Dawes

**Affiliations:** 1 Center for Physiology, Pathophysiology and Immunology, Institute of Physiology, Section of Physiology of Lipid Metabolism, Medical University of ViennaVienna, Austria; 2 Department of Biochemistry and Biocenter Oulu, University of OuluOulu, Finland; 3 Max F. Perutz Laboratories, Department of Biochemistry, University of ViennaVienna, Austria; 4 School of Biotechnology and Biomolecular Sciences, University of New South WalesSydney, NSW, Australia

**Keywords:** *Saccharomyces cerevisiae*, oleic acid induction, oleate response element ORE, midsporulation element (MSE), Sum1p repressor, Hst1p, Rfm1p

## Abstract

The sporulation-specific gene *SPS18* shares a common promoter region with the oleic acid-inducible gene *SPS19*. Both genes are transcribed in sporulating diploid cells, albeit unevenly in favour of *SPS18*, whereas in haploid cells grown on fatty acids only *SPS19* is highly activated. Here, *SPS19* oleate-response element (ORE) conferred activation on a basal *CYC1-lacZ* reporter gene equally in both orientations, but promoter analysis using *SPS18-lacZ* reporter constructs with deletions identified a repressing fragment containing a midsporulation element (MSE) that could be involved in imposing directionality towards *SPS19* in oleic acid-induced cells. In sporulating diploids, MSEs recruit the Ndt80p transcription factor for activation, whereas under vegetative conditions, certain MSEs are targeted by the Sum1p repressor in association with Hst1p and Rfm1p. Quantitative real-time PCR demonstrated that in haploid *sum1*Δ, *hst1*Δ, or *rfm1*Δ cells, oleic acid-dependent expression of *SPS18* was higher compared with the situation in wild-type cells, but in the *sum1*Δ mutant, this effect was diminished in the absence of Oaf1p or Pip2p. We conclude that *SPS18* MSE is a functional element repressing the expression of both *SPS18* and *SPS19*, and is a component of a stricture mechanism shielding *SPS18* from the dramatic increase in ORE-dependent transcription of *SPS19* in oleic acid-grown cells.

## Introduction

Divergent genes occur as two ORFs, one on each DNA strand, that are transcribed outwardly from a common promoter region delineated by the pair's ATG start sites. The compact genome of the yeast *Saccharomyces cerevisiae* contains numerous divergent genes, which, in certain cases, are involved in the same cellular process. Simultaneous regulation of metabolically linked divergent genes is mediated by promoter elements that direct the transcriptional machinery towards each of the coding sequences. For example, *GAL1* and *GAL10* required for galactose breakdown are coordinated by UAS_G_ located between the genes (Johnston & Davis, 1984; West *et al.*, 1984; Yocum *et al.*, 1984;).

Other divergent systems, such as the one represented by the two sporulation-specific genes *DIT1* and *DIT2*, use repressors for coordinated control. In this case, a *DIT* repressor element constituting the major negative regulatory site during vegetative growth (Bogengruber *et al.*, 1998) exerts repression in conjunction with a midsporulation element (MSE) situated within an negative regulatory element (NRE_DIT_) (Friesen *et al.*, 1997). However, at least from the probable functions assigned to them in the yeast databases (YPD™, http://www.proteome.com; SGD™, http://genome-www.stanford.edu), it seems that in the vast majority of cases divergent genes encode proteins that are not involved in the same process.

Divergent genes could ostensibly be regulated from their common promoter region through elements operating unidirectionally to enhance transcription of only one gene at a time. In general, however, known regulatory transcription factors targeting promoter elements act in both orientations (Angermayr & Bandlow, 1997). This feature also holds true for the two-tracked activating mechanism in the respective promoters of the *POT1/FOX3* and *CTA1* genes that are induced on oleic acid medium (Einerhand *et al.*, 1993; Filipits *et al.*, 1993;). Hence, to ensure temporal specificity, divergent gene systems that are guided by different transcriptional schedules must use dedicated stricture mechanisms.

**Fig. 1:**
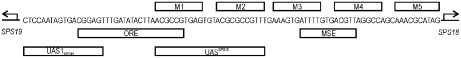
Scheme of the *SPS18–SPS19* intergenic region incorporated into the reporter genes used. A 1.4-kb XbaI–SphI fragment including the shared *SPS18–SPS19* promoter region and a portion of the reading frames of both genes was used as template for site-directed mutagenesis. The distance between the two ATG translational start codons is 300 bp. The terminal 3′ G in the depicted sequence occurs 79 nucleotides upstream of the *SPS18* ATG site, whereas the terminal 5′ C is 130 bp upstream of the *SPS19* ATG triplet. The sequences representing UAS1_SPS19_, *SPS19* ORE, UAS^SPS19^, and *SPS18* MSE, are indicated as boxes below the sequence. Boxed regions above the sequence represent mutations introduced into the promoter that was incorporated within the various reporter genes used. An XhoI restriction site (CTCGAG) was substituted for the boxed DNA sequences designated M1 and M3, whereas regions designated M2, M4, and M5 were deleted. TATA-box sequences TATAAA or TATAAG occur 61 and 103 nucleotides 5′ of the *SPS18* and *SPS19* ATG start codons, respectively.

The present study is concerned with the manner in which divergent gene promoters containing bidirectional elements mediate selective regulation in only one orientation. The *SPS18–SPS19* gene pair was chosen as a test system because previous work demonstrated that despite being separated by a short 300-nucleotide promoter region, their individual transcription schedules vary significantly. For example, in diploids undergoing sporulation, *SPS18* is highly transcribed between 5 and 11 h into the process, whereas *SPS19* transcription is very much lower (Coe *et al.*, 1994; Chu *et al.*, 1998;). In haploids grown under oleic acid medium conditions, *lacZ* reporter genes in combination with Northern blotting revealed that transcription of *SPS19* is over 25-fold higher than *SPS18* (Gurvitz *et al.*, 1997a,b;). This activation occurs at a resident oleate-response element (ORE) in combination with overlapping UAS^SPS19^ (Gurvitz *et al.*, 1999) and UAS1_SPS19_ (Gurvitz *et al.*, 2000) components (Fig. 1; boxes below the DNA sequence), the latter binding the transcription factor Adr1p (Eisen *et al.*, 1988). Adr1p is important for cell growth under derepressing conditions, and is required for transcribing *SPS19* but not *SPS18* (Young *et al.*, 2003; Karpichev *et al.*, 2008;).

The *SPS18–SPS19* promoter region also contains a functional MSE (Fig. 1) that responds to the transcription factor Ndt80p (Ozsarac *et al.*, 1997; Chu *et al.*, 1998;). Certain MSEs additionally bind Sum1p – in combination with Hst1p (Xie *et al.*, 1999; Pierce *et al.*, 2003;) and Rfm1p (McCord *et al.*, 2003) – to repress genes under vegetative conditions. Indeed, both *SPS18* and *SPS19* are upregulated in the absence of Hst1p, but their unscheduled expression profiles do not resemble each other (Wyrick *et al.*, 1999). To elucidate the mechanism repressing *SPS18* when expression of *SPS19* is induced, a set of deletions in the promoter region was constructed and their effect on *SPS18* expression was determined. The action of Sum1p, Hst1p and Rfm1p on the *SPS18*–*SPS19* intergenic region under oleic acid-induction conditions was also assessed. The results are discussed in terms of the shielding of genes in divergent systems from unscheduled transcriptional activation.

## Materials and methods

### Strains, plasmids, and oligonucleotides

The *S. cerevisiae* strains and plasmids used are listed in Tables 1 and 2, respectively. The *Escherichia coli* strain DH10B was used for all plasmid amplifications and isolations. Construction of the BJ1991-derived strains (Jones, 1977), BJ1991*pip2*Δ, BJ1991*oaf1*Δ, and BJ1991*pip2*Δ*oaf1*Δ (Rottensteiner *et al.*, 1996, 1997) or yAG259 and yAG561 (Gurvitz *et al.*, 1997b), has been described. To generate strains yAG547, yAG554, yAG565, yAG557, and yAG569, the respective plasmids pAG528, pAG530, pAG536, pAG532, or pAG538 were linearized using StuI and verifiably integrated as a single copy (Southern, 1975) into the *ura3* locus of BJ1991 wild-type (WT) cells (Chen *et al.*, 1992). Strains yAG1310 and yAG1312 were constructed by integrating a single copy of StuI-linearized plasmids pAG534 or pAG536, respectively, into the *ura3* locus of BJ1991*pip2*Δ*oaf1*Δ. Strain yHPR1550 was constructed by introducing a single copy of a StuI-linearized pSPS19 ORE:CYC1-lacZ plasmid into the *ura3* locus of BJ1991 WT cells. The WT strain BY4741 and its *sum1*Δ, *hst1*Δ, and *rfm1*Δ derivatives were obtained from EUROSCARF (http://www.uni-frankfurt.de). Strains yAG1193 and yAG1230 were constructed by inserting a single copy of a StuI-linearized plasmid pAG534 into the *ura3* loci of strains BY4741 WT and BY4741*sum1*Δ. BY4741-based *pip2*Δ, *oaf1*Δ, *sum1*Δ*pip2*Δ, or *sum1*Δ*oaf1*Δ mutants were constructed by integrating into the *leu2* locus of the respective parental strains the *pip2*Δ*::LEU2* fragment generated from an SpeI- and NcoI-digested pSKΔPIP2 plasmid (Rottensteiner *et al.*, 1996) or the *oaf1*Δ*::LEU2* fragment produced by digesting plasmid pAK83 with NcoI and HindIII (Rottensteiner *et al.*, 1997).

**Table 2:** Plasmids and oligonucleotides used

	Description	Sources or references
Plasmids		
pSPS19ORE-CYC1-lacZ	*SPS19* ORE:*CYC1-lacZ*, fusion boundary towards *SPS19*	This study
pAG244	*SPS19* ORE:*CYC1-lacZ*, fusion boundary towards *SPS18*	Gurvitz *et al.* (1997b)
pMF6	Integrative plasmid vector for the above two reporter genes	Filipits *et al.* (1993)
YIp356R/YIp357	*URA3*-marked integrative vectors for *lacZ* fusions	Myers *et al.* (1986)
pAG534	*SPS18–lacZ*; YIp356R with 1.4-kb *SPS18/19* fragment	Gurvitz *et al.* (1997b)
pAG528	As above but with an XhoI-site substitution at M1	A. Gallagher, UNSW
pAG530	As above but with a deletion at M2	A. Gallagher, UNSW
pAG536	As above but with an XhoI-site substitution at M3	This study
pAG532	As above but with a deletion at M4	A. Gallagher, UNSW
pAG538	As above but with a deletion at M5	This study
pSKΔPIP2	*pip2*Δ::*LEU2* disruption plasmid	Rottensteiner *et al.* (1996, 1997)
pAK83	*oaf1*Δ::*LEU2* disruption plasmid	Rottensteiner *et al.* (1997)
Oligonucleotides		
ACT1-928F	GCCGAAAGAATGCAAAAGGA	This study
ACT1-1001R	TCTGGAGGAGCAATGATCTTGA	This study
SMK1-988F	CAAGCTATATCAC ATCCGTTCCTAAA	This study
SMK1-1060R	AAGGACCCTGAAGGCAAACA	This study
SPS18-790F	ATCAAGAGATCATTCGTGCACTTTA	This study
SPS18-862R	AAGAAAAAACTGGCGAGGGTAA	This study
SPS19-331F	GCCGGTGCTGCTGGAA	This study
SPS19-399R	AACAACAGATTTGAAGGCGTTTG	This study

**Table 1:** *Saccharomyces cerevisiae* strains used

	Description	Sources or references
Strains		
(1) BJ1991	*MAT*α*leu2 ura3-52 trp1 pep4-3 prb1-122 gal2*	Jones (1977)
(2) BJ1991*pip2*Δ^1*^	*pip2*Δ::*KanMX4*	Rottensteiner *et al.* (1997)
(3) BJ1991*oaf1*Δ^1^	*oaf1*Δ::*LEU2*	Rottensteiner *et al.* (1997)
yHPR1550^1^	pSPS19 ORE:CYC1-lacZ	This study
yAG259^1^	pAG244 (*SPS19* ORE::CYC1-lacZ)	Gurvitz *et al.* (1997b)
yAG561^1^	pAG534 (*SPS18–lacZ* WT)	Gurvitz *et al.* (1997b)
yAG547^1^	pAG528 (*SPS18–lacZ* M1)	This study
yAG554^1^	pAG530 (*SPS18–lacZ* M2)	This study
yAG565^1^	pAG536 (*SPS18–lacZ* M3)	This study
yAG557^1^	pAG532 (*SPS18–lacZ* M4)	This study
yAG569^1^	pAG538 (*SPS18–lacZ* M5)	This study
(4) BJ1991*pip2*Δ*oaf1*Δ^1^	*pip2*Δ::*KanMX4 oaf1*Δ::*LEU2*	Rottensteiner *et al.* (1997)
yAG1310^4^	pAG534 (*SPS18–lacZ* WT)	This study
yAG1312^4^	pAG536 (*SPS18–lacZ* M3)	This study
(5) BY4741	*MAT***a***his3*Δ*1 leu2*Δ*0 met15*Δ*0 ura3*Δ*0*	EUROSCARF
(6) BY4741*sum1*Δ^5^	*YDR310c*::*kanMX4*	EUROSCARF
BY4741*hst1*Δ^5^	*YOL068c*::*kanMX4*	EUROSCARF
BY4741*rfm1*Δ^5^	*YOR279c*::*kanMX4*	EUROSCARF
yAG1193^5^	expressing *SPS18–lacZ* from pAG534	This study
yAG1230^6^	expressing *SPS18–lacZ* from pAG534	This study
BY4741*oaf1*Δ^5^	*oaf1*Δ::*LEU2* from pAK83	This study
BY4741*pip2*Δ^5^	*pip2*Δ::*LEU2* from pSKPΔPIP2	This study
BY4741*sum1*Δ*oaf1*Δ^6^	*oaf1*Δ::*LEU2* from pAK83	This study
BY4741*sum1*Δ*pip2*Δ^6^	*pip2*Δ::*LEU2* from pSKΔPIP2	This study

* The numbers in superscript following the strains' designation refer to their parental genotypes; for example, BJ1991*pip2*Δ^1^ was derived from (1) BJ1991.

### Plasmid constructions

Nucleic acids were manipulated as described (Sambrook *et al.*, 1989). Construction of integrative plasmids with promoters containing deletions was described previously (Gurvitz *et al.*, 1997b). Briefly, a 1.4-kb XbaI–SphI fragment containing the intergenic region and part of the coding regions of *SPS19* was excised from pUC18-KXC (Coe *et al.*, 1994) and inserted into M13mp19 for either deletion of the promoter regions M3, M4, and M5, or substitution at M1 and M3 with a unique XhoI site, using site-directed mutagenesis. The mutated DNA was verified by nucleotide sequencing. Construction of plasmid pAG534 containing the WT *SPS18* promoter fused with the *lacZ* gene in YIp356R (Myers *et al.*, 1986) was outlined previously (Gurvitz *et al.*, 1997b). Plasmids pAG528, pAG530, and pAG532 consisted of the respective M1, M2, and M4 mutated promoters. Plasmids pAG536 and pAG538 (M3 and M5 mutated promoters, respectively) were constructed here. Plasmid pSPS19 ORE:CYC1-lacZ was constructed from pMF6 (Filipits *et al.*, 1993) essentially as described for pAG244 (Gurvitz *et al.*, 1997b).

### Media and growth conditions

Standard yeast (Rose *et al.*, 1990) and *E. coli* (Sambrook *et al.*, 1989) media were made as described. *Saccharomyces cerevisiae* strains were propagated on solid rich-glucose YPD medium consisting of 1% (w/v) yeast extract – 2% (w/v) peptone (YP), 2% (w/v) d-glucose, and 2% (w/v) agar. Selection for integrative or disruption plasmids in transformed strains was carried out using solid synthetic defined (SD) medium consisting of 0.67% (w/v) yeast nitrogen base without amino acids, 2% (w/v) d-glucose, 3% (w/v) agar, with all supplements added except for uracil (SD-Ura) or leucine (SD-Leu).

Liquid oleic acid medium (YPO) consisted of YP, 0.05% (w/v) glucose, 0.2% (w/v) oleic acid and 0.02% (w/v) Tween 80, adjusted to pH 7 with NaOH (Gurvitz *et al.*, 1997b). For β-galactosidase measurements using *o*-nitrophenyl-β-d-galactopyranoside (ONPG) (Miller, 1972; Rottensteiner *et al.*, 1996;), cells were induced in YPO medium as follows: late log-phase cells from overnight YPD precultures were transferred to 100-mL conical flasks containing 50 mL YPO (with 75 μg mL^−1^ ampicillin) to A_600 nm_=0.2. The cultures were returned to shaking and samples were removed for analysis at the indicated times. Protein concentrations were determined using the BioRad dye (Bradford, 1976).

### RNA isolation

Triplicate cultures of *S. cerevisiae* cells induced in YPO were collected by centrifugation (3000 ***g*** at 4 °C for 5 min), washed twice in two volumes of cold distilled water, and frozen in liquid nitrogen. RNA samples were extracted with the Master Pure™ Yeast RNA Purification Kit (Epicentre Biotechnologies, WI) according to the manufacturer's instructions. Following isolation, RNA was treated twice with an RNAse-Free DNAse set (Qiagen, Hilden, Germany) in keeping with supplier instructions. To verify the removal of contaminating genomic DNA, RNA samples were subjected to thermocycling amplification without reverse transcriptase.

### Quantitative real-time PCR and data analysis

Total RNA (5 μg) was processed using reverse transcriptase into first-strand cDNA in 20-μL reactions with RevertAid™ First Strand cDNA Synthesis Kit (Fermentas, Helsinki, Finland). To generate primers for real-time PCR, the nucleotide sequences of the *S. cerevisiae* genes *SPS18, SPS19*, *SMK1*, and *ACT1* were scrutinized using the primer express software (Applied Biosystems, Foster City, CA), and the oligonucleotides were supplied by Sigma-Aldrich Inc. in the United Kingdom. Real-time PCR was undertaken with an ABI PRISM 7000 sequence detector and analysed using the abi prism 7500 sequence detector software v. 1.4 (Applied Biosystems). Amplification was carried out in 30-μL reaction mixtures consisting of 1 × SYBR Green PCR master mix (Applied Biosystems), 4.5 nL of cDNA reaction mixture and 2 pmol μL^−1^ primer sets. Thermocycling was performed in 40 cycles of a two-step PCR (95 °C for 15 s and 60 °C for 1 min) after an initial activation (95 °C for 10 min) of DNA polymerase. A heat dissociation protocol was applied to the PCR reactions to ensure that the SYBR green dye detected only one PCR product. Triplicate cDNAs from each sample were amplified using primers for *SPS18, SPS19*, *SMK1*, and *ACT1* genes. Two independent assays with the same cDNA samples and primers for *SPS18, SPS19*, *SMK1*, and *ACT1* were undertaken and values were measured for each individual experiment. Following SYBR Green PCR amplification, data acquisition and subsequent data analyses were carried out using the abi prism 7500 sequence detector software 1.4. The PCR cycle at which a statistically significant increase in the Δ*R_n_* (the fluorescence of SYBR Green relative to that of internal passive dye, ROX) is first detected is called the threshold cycle (*C*_t_). The Δ*C*_t_ refers to the difference between the mean *C*_t_ value of the *SPS18, SPS19*, *SMK1*, and the endogenous control, *ACT1*. The ΔΔ*C*_t_ represents the difference between the mean Δ*C*_t_ value of the calibrator BY4741 WT culture and the corresponding mutant strains (Table 1). The amount of target, normalized to an endogenous reference and relative to a calibrator, is given by 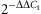. Derivation of the 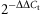 equation has been described in Applied Biosystems, User Bulletin No. 2 (P/N 4303859). Hence, experimental samples could be expressed as an *n*-fold difference relative to the calibrator. For the real-time assays with the 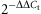 method, the amplification efficiency of the target gene and internal control gene was tested by plotting the amount of the input template vs. the Δ*C*_t_, where a slope of *c*. 0 demonstrates that the efficiencies were comparable.

## Results

### *SPS19* ORE mediates bidirectional transcription

Cells propagated in oleic acid medium do not transcribe *SPS18* to the same level as the ORE-dependent gene *SPS19* (Gurvitz *et al.*, 1997a,b;). *SPS19* ORE complies with the consensus sequence CGGN_3_TN^A^/_R_N_8–12_CCG (Gurvitz & Rottensteiner, 2006), which binds the Pip2p-Oaf1p transcription factor (Luo *et al.*, 1996; Rottensteiner *et al.*, 1996;). Although the OREs in the promoters of the *POT1/FOX3* and *CTA1* genes (Einerhand *et al.*, 1993; Filipits *et al.*, 1993;) have been shown to confer bidirectional transcription on a basal *CYC1* promoter, it was not clear from the outset whether the *SPS19* ORE acts equally in both directions.

To examine whether *SPS19* ORE intrinsically activates transcription with a preference towards *SPS19*, the element was tested for orientation bias in conferring transcription on a basal *CYC1* promoter. Cells expressing a *CYC1-lacZ* reporter gene in which *SPS19* ORE was inserted in either orientation were monitored following 18-h growth on oleic acid. The results demonstrated similar levels of β-galactosidase activity irrespective of insert orientation (*c*. 20-fold greater than at 0 h; Table 3). This indicated that the minimal sequence of *SPS19* ORE did not contain additional information relating to orientation of transcription, albeit nucleotides within the ORE might act in conjunction with neighbouring sequences to effect unidirectionality, such as in the situation with UAS^SPS19^ (Gurvitz *et al.*, 1999), in which a mild degree of direction is enforced on the 5′-ORE half site. Therefore, a further stricture or boundary mechanism must exist that confines the regulatory action of the oleic acid-specific *trans*-activator Pip2p-Oaf1p to transcribing *SPS19*.

**Table 3:** The effect of inserting *SPS19* ORE in either orientation on the transcription of an integrative basal *CYC1-lacZ* reporter construct in haploid cells following oleic acid induction

		β-Galactosidase activity*	
Strains	Direction of *lacZ* fusion	0 h	18 h†
yHPR1550	Towards *SPS19*	11	221
yAG259	Towards *SPS18*	7	225

* nmol ONPG metabolized min^−1^ mg^−1^ protein.

† Performed in duplicates.

### Loss of *SPS18* repression

To analyse the *SPS18–SPS19* intergenic region for sequences that might be involved in throttling the transcription of *SPS18* during oleic acid induction, a set of strains was generated harbouring *lacZ* reporter genes carrying the WT promoter or a promoter containing deleted segments (M1 through M5; Fig. 1). Levels of β-galactosidase expression by *SPS18–lacZ* were measured in soluble protein extracts from cells grown overnight on rich-glucose medium (0 h) followed by 18 h propagation on oleic acid. The results demonstrated that the precultures at 0 h with the M1–M4 reporter genes gave rise to levels of β-galactosidase activities that were higher compared with the WT construct (Table 4), although these values were at the lower detection limit of the method used. Following 18-h growth on oleic acid medium, a decreased level of β-galactosidase activity was recorded for the M1 reporter gene compared with the WT, which coincided with perturbed ORE and UAS^SPS19^ elements in the promoter of the mutant construct (Fig. 1; Table 4). On the other hand, mutant reporter genes M2–M5 gave rise to activities that were at least twofold higher compared with those obtained using the WT construct (Table 4). This indicated the loss of a potential repressor element (an operator site). For comparison, a previous experiment conducted on the same set of mutations, but in the settings of an *SPS19–lacZ* reporter, gave the following values: WT, 1 (relative level); M1, 0.01; M2, 0.29; M3, 0.85; M4, 0.92, and M5, 0.67 (Gurvitz *et al.*, 1999). The M3–M4 demarcated region overlaps an MSE (Ozsarac *et al.*, 1997) that could turn out to be a repressor element of the *SMK1-NHP6A* type (Xie *et al.*, 1999). Hence, loss of the MSE repressor element could lead to (1) a more active basal promoter, (2) a misdirection of ORE-dependent transcriptional activation towards *SPS18*, or (3) a combination of both. Were the observed unscheduled transcription of *SPS18* shown to be subordinate to Pip2p-Oaf1p, this could help elucidate the cause of this effect.

**Table 4:** The effect of deletions in the *SPS18–SPS19* promoter on the expression of an integrative *SPS18–lacZ* reporter gene in haploid cells grown under oleic acid medium conditions

			β-Galactosidase activity*			
Strains	Reporters	Mutated at	0 h†	18 h‡	Fold induction	Relative level
yAG561	WT	Not mutated	4	15 ± 1	3.8 ×	1.0
yAG547	M1	ORE, UAS^SPS19^	5	8 ± 3	1.6 ×	0.6
yAG554	M2	UAS^SPS19^	10	42 ± 6	4.2 ×	2.8
yAG565	M3	*SPS18* MSE	5	38 ± 6	7.6 ×	2.6
yAG557	M4	*SPS18* MSE	7	43 ± 8	6.1 ×	2.9
yAG569	M5	3′ to MSE	3	31 ± 12	10.3 ×	2.0

* nmol ONPG metabolized min^−1^ mg^−1^ protein.

† Performed in duplicates.

‡ Mean ± SD; *n*=6.

### *SPS18* MSE represses *SPS18* transcription on glucose and oleic acid

The *SMK1-NHP6A* MSE blocks the expression of the former gene from the constitutive transcription of the latter, thereby representing a repressor element (Xie *et al.*, 1999). In the case of *SPS18*, however, the divergent *SPS19* gene is not constitutively expressed, but instead is subordinate to an ORE that induces transcription by over 20-fold in response to oleic acid (Gurvitz *et al.*, 1997a,b;). The *SPS19* ORE palindrome is overlapped by other elements at each half site, an Adr1p-binding element UAS1_SPS19_ at its 3′-half site, and a separate UAS^SPS19^ at the other. *SPS19* transcription fails to become induced in the absence of either the Pip2p-Oaf1p or Adr1p transcription factors that have been shown to bind *SPS19* ORE and UAS1_SPS19_, respectively.

If loss of the MSE leads to higher basal activity of *SPS18*, then we would predict that the level of transcription would not depend on the carbon-source responsiveness of the Pip2p-Oaf1p activator complex. On the other hand, were the *SPS18* MSE to shield *SPS18* in cells grown on oleic acid from the high levels of ORE-dependent transcriptional activation of *SPS19*, unscheduled transcription of *SPS18* would be subordinate to Pip2p-Oaf1p. To examine which of the two possible scenarios predominates, the previously used *SPS18–lacZ* reporter genes WT and the MSE-less M3 were examined in WT cells as well as a *pip2*Δ*oaf1*Δ mutant strain (Karpichev *et al.*, 1997; Rottensteiner *et al.*, 1997;) in which transcriptional activation of *SPS19* is abrogated (Gurvitz *et al.*, 1997a). Both reporter genes contain an intact ORE; Pip2p-Oaf1p is not known to interact with MSEs.

**Fig. 2:**
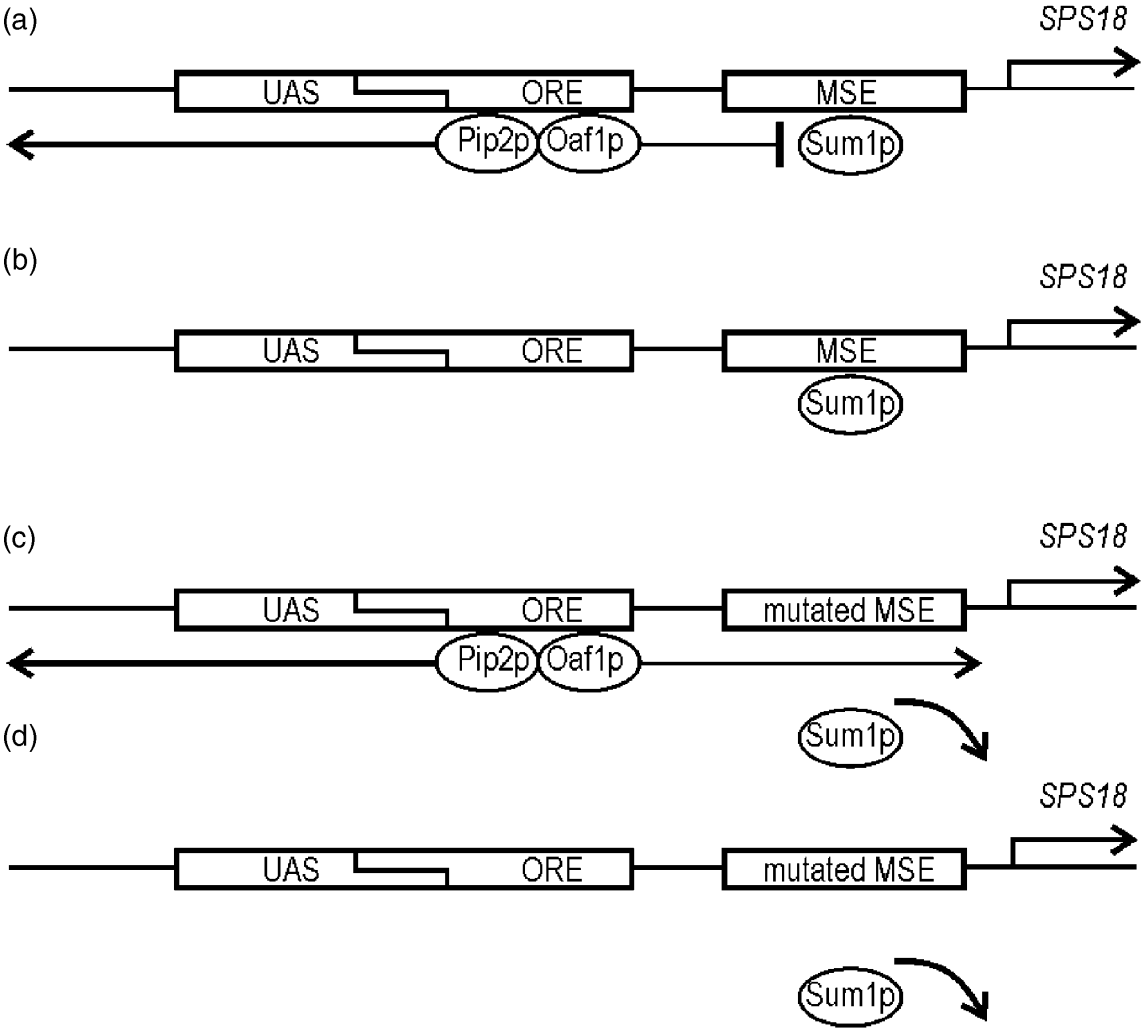
Scheme of the promoter arrangement in the strains reported in Table 5. In the WT strain yAG561 expressing the WT reporter gene (a), Sum1p acting at the MSE is proposed to block oleic acid-induced transcriptional activation (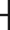) due to Pip2p-Oaf1p binding to the ORE, so that no decrease in reporter-gene expression levels was etected when Pip2p-Oaf1p was absent in the mutant strain yAG1310 (b). However, when the MSE was mutated within the M3 reporter construct (c) and Sum1p could not act on its cognate element, Pip2p-Oaf1p could induce transcription of *SPS18* (→) in the WT strain yAG565 beyond the levels attained by the *pip2*Δ*oaf1*Δ mutant yAG1312 (d). Thick and thin arrows indicate high or low levels of transcription, respectively. UAS refers to an overlapping Adr1p-binding element.

The results demonstrated that levels of β-galactosidase activities expressed from the M3-reporter gene in the WT strain yAG565 following overnight growth in rich-glucose medium (0 h) were slightly higher than from the WT reporter in the WT strain yAG561 grown under similar conditions (3 U compared with 2U; Table 5). Notwithstanding the fact that these 0-h measurements of the precultures were at the detection limit, this indicated that the basal activity of the promoter might have been higher in the reporter construct lacking a complete MSE. Following 18-h oleic acid-medium conditions, β-galactosidase activities from the M3 fusion in yAG565 were *c*. 2.5-fold higher than those from the WT fusion in yAG561 (27 U compared with 11 U; Table 5), and almost twofold higher compared with the *pip2*Δ*oaf1*Δ mutant yAG1312 (27 U compared with 15 U; Table 5), whereas expression levels of the parental WT reporter gene in both the WT strain yAG561 as well as in the *pip2*Δ*oaf1*Δ mutant yAG1310 were essentially identical, at about 11 U. Hence, from the results presented here, it emerged that in addition to repressing *SPS18* transcription under vegetative conditions, the MSE also appeared to play a role in shielding *SPS18* from unscheduled ORE-dependent transcription (Fig. 2). Confirmation of these reporter-gene results was undertaken using quantitative real-time PCR.

**Table 5:** **.**The effect of deleting *PIP2* and *OAF1* on the expression of WT and M3 *SPS18–lacZ* reporter genes in haploid cells grown under oleic acid-medium conditions

			β-Galactosidase activity*			
Strains			0 h	18 h†	Fold induction	Relative level
WT reporter (intact MSE)						
A‡	yAG561	WT	2	11	5.5 ×	1.0
B	yAG1310	*pip2*Δ*oaf1*Δ	1	11	11.0 ×	1.0
M3 reporter (mutated MSE)						
C	yAG565	WT	3	27 ± 1	9.0 ×	2.5
D	yAG1312	*pip2*Δ*oaf1*Δ	3	15 ± 1	5.0 ×	1.3

* nmol ONPG metabolized min^−1^ mg^−1^ protein.

† Mean ± SD; *n*=3.

‡ Refers to the element arrangement in Fig. 2.

### *SPS18* MSE relies on Sum1p for repressing *SPS18*

MSEs represent the target for the sporulation-specific transcription factor Ndt80p (Xu *et al.*, 1995; Chu *et al.*, 1998;). In addition, MSEs are also the target for Sum1p – in association with Hst1p and Rfm1p (Xie *et al.*, 1999; McCord *et al.*, 2003;), which act in unison to repress certain midsporulation genes under vegetative conditions (Xie *et al.*, 1999). To determine whether Sum1p is important for repressing *SPS18*, a single copy of the WT *SPS18–lacZ* reporter gene was introduced into the genome of a WT BY4741 haploid strain as well as into that of an otherwise isogenic mutant with a deletion in the *SUM1* gene.

**Fig. 3:**
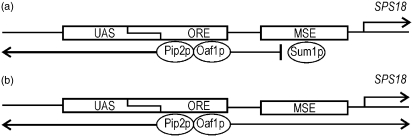
Scheme of the promoter arrangement in the strains reported in Table 6. In the WT strain yAG1230 expressing the WT *SPS18–lacZ* reporter construct (a), Sum1p could act at the MSE to block oleic acid-induced transcriptional activation (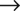) due to Pip2p-Oaf1p binding to the ORE, but in yAG1193 cells devoid of Sum1p (b); this latter activation proceeded unhindered (→), resulting in higher levels of reporter-gene activity. The thick arrows reflect higher levels of transcription compared with those depicted by the thin arrows. The Adr1p-binding element overlapping the ORE is referred to as UAS.

The results of the reporter-gene assays performed on these two strains (Table 6) showed that following an 18-h propagation on oleic acid medium, β-galactosidase activities in the WT harbouring *SPS18–lacZ* increased 1.6-fold compared with glucose, whereas in the *sum1*Δ mutant this increase was 3.7-fold. On oleic acid, *SPS18–lacZ* was 2.9-fold more highly expressed in the *sum1*Δ mutant than in the WT (116 vs. 40 U) as compared with a 1.2-fold increase in activity between these two strains when grown on glucose (31 vs. 25 U). Hence, Sum1p appeared to shield *SPS18* from oleic acid-induced transcription activation (Fig. 3), and this could be exposed using quantitative real-time PCR.

**Table 6:** The effect of mutating *SUM1* on *SPS18–lacZ* reporter gene expression following an 18-h oleic acid induction of haploid cells

			β-Galactosidase activity*			
Strains			0 h	18 h†	Fold induction	Relative level
A‡	yAG1230	WT	25	40 ± 3	1.6 ×	**1.0**
B	yAG1193	*sum1*Δ	31	116 ± 5	3.7 ×	2.9

* nmol ONPG metabolized min^−1^ mg^−1^ protein.

† Mean ± SD; *n*=3.

‡ Refers to the element arrangement in Fig. 3.

### Quantitative real-time PCR of the roles of Sum1p/Hst1p/Rfm1p in repressing *SPS18*

To determine the physiological levels of *SPS18* transcripts when the MSE is not occupied by Sum1p or its two associates, quantitative real-time PCR was performed. Yeast cells were propagated in liquid oleic acid medium for 18 h, and following cell breakage, RNA was extracted (with the concomitant removal of contaminating DNA) in order to provide template for thermocycling amplification, which was applied to *SPS18* and *SPS19*. As a positive control for the effect of Sum1p, *SMK1* was also primed for amplification. In addition, expression of the gene for actin, *ACT1*, which is not upregulated in yeast grown on oleic acid, was also monitored.

The results in Table 7 demonstrated that in the WT haploid strain, the threshold cycle of *SPS19* preceded that of *SPS18* by almost 10 cycles, and hence the former's expression was considerably higher (850-fold compared with *SPS18*). In addition, the influence of the three deletions on the control *SMK1* gene verified their physiological effect on releasing repression from the MSE (1.6–3.3-fold). Moreover, these deletions also increased *SPS18* expression by 7.1–9.6-fold when compared with the WT, confirming the observations made with *SPS18–lacZ* reporter gene in Table 6. A lower effect on elevating *SPS19* expression in these mutants was also noted (2.1–2.5-fold).

**Table 7:** Real-time PCR revealing the effect of deleting *SUM1, HST1*, or *RFM1* on *SPS19* and *SPS18* expression in haploid cells following an 18-h oleic acid induction

Strains	Genes	Average *C*_t_*	Average Δ*C*_t_± SE	ΔΔ*C*_t_	RQ
WT	*SPS18*	33.120	11.089 ± 0.222	0	1
	*SPS19*	23.388	1.357 ± 0.510	0	1
	*SMK1*	29.961	7.930 ± 0.243	0	1
	*ACT1*	22.031			
*sum1*Δ	*SPS18*	29.855	7.831 ± 0.234	−3.258	9.564
	*SPS19*	22.282	0.258 ± 0.147	−1.099	2.142
	*SMK1*	29.275	7.252 ± 0.267	−0.678	1.600
	*ACT1*	22.024			
*hst1*Δ	*SPS18*	30.620	8.272 ± 0.039	−2.817	7.047
	*SPS19*	22.673	0.325 ± 0.046	−1.032	2.045
	*SMK1*	28.748	6.401 ± 0.114	−1.529	2.887
	*ACT1*	22.348			
*rfm1*Δ	*SPS18*	31.176	8.120 ± 0.091	−2.969	7.829
	*SPS19*	23.080	0.025 ± 0.083	−1.333	2.519
	*SMK1*	29.263	6.208 ± 0.158	−1.722	3.300
	*ACT1*	23.056			

* In this and the ensuing Tables 8 and 9, the significance of *C*_t_, Δ*C*_t_, and ΔΔ*C*_t_ is explained in Materials and methods.

RQ, relative quantity.

**Table 9:** Real-time PCR displaying the consequences to *SPS19* and *SPS18* expression of deleting both *SUM1* and *PIP2* following an 18-h oleic acid induction of haploid cells

Strains	Genes	Average *C*_t_	Average Δ*C*_t_± SE	ΔΔ*C*_t_	RQ
WT	*SPS18*	31.972	10.637 ± 0.24	0	1
	*SPS19*	21.528	0.367 ± 0.154	0	1
	*SMK1*	27.089	5.851 ± 0.21	0	1
	*ACT1*	21.21			
*pip2*Δ	*SPS18*	33.042	11.852 ± 0.118	1.215	0.431
	*SPS19*	25.203	3.661 ± 0.041	3.294	0.102
	*SMK1*	27.314	5.811 ± 0.078	−0.04	1.028
	*ACT1*	21.519			
*sum1*Δ	*SPS18*	29.334	7.711 ± 0.044	−2.926	7.598
	*SPS19*	20.961	−0.584 ± 0.084	−0.951	1.934
	*SMK1*	27.316	5.795 ± 0.096	−0.056	1.04
	*ACT1*	21.536			
*sum1*Δ*pip2*Δ	*SPS18*	30.779	8.979 ± 0.141	−1.658	3.155
	*SPS19*	23.038	1.31 ± 0.026	0.943	0.52
	*SMK1*	27.279	5.515 ± 0.033	−0.336	1.262
	*ACT1*	21.766			

RQ, relative quantity.

**Table 8:** The effect of mutating both *SUM1* and *OAF1* on *SPS19* or *SPS18* expression following an 18-h oleic acid induction of haploid cells, as shown by real-time PCR

Strains	Genes	Average *C*_t_	Average Δ*C*_t_± SE	ΔΔ*C*_t_	RQ
WT	*SPS18*	28.701	6.369 ± 0.415	0	1
	*SPS19*	22.432	0.101 ± 0.425	0	1
	*SMK1*	30.164	7.832 ± 0.39	0	1
	*ACT1*	22.331			
*oaf1*Δ	*SPS18*	31.573	9.419 ± 0.383	3.049	0.121
	*SPS19*	27.651	5.497 ± 0.121	5.396	0.024
	*SMK1*	30.1	7.946 ± 0.293	0.114	0.924
	*ACT1*	22.154			
*sum1*Δ	*SPS18*	28.908	4.118 ± 0.916	−2.251	4.762
	*SPS19*	24.793	0.003 ± 0.976	−0.098	1.07
	*SMK1*	31.873	7.083 ± 0.985	−0.749	1.681
	*ACT1*	24.79			
*sum1*Δ*oaf1*Δ	*SPS18*	29.109	6.552 ± 0.499	0.183	0.881
	*SPS19*	25.45	2.893 ± 0.655	2.793	0.144
	*SMK1*	29.943	7.386 ± 0.408	−0.446	1.362
	*ACT1*	22.556			

RQ, relative quantity.

To determine whether the increase in *SPS18* expression in the *sum1*Δ mutant was due, at least in part, to oleic acid-dependent induction, *sum1*Δ*oaf1*Δ and *sum1*Δ*pip2*Δ double mutants were generated and examined for their ability to express *SPS18* and *SPS19*. The rationale behind this experiment was that if *SPS18* expression in the double mutants was lower than in the parental *sum1*Δ mutant (as would be expected for the Pip2p- and Oaf1p-dependent gene *SPS19*), this would indicate that in *sum1*Δ cells loss of MSE function allowed oleic acid-dependent transcription to proceed in the wrong orientation.

The results in Table 8 showed that in the WT strain, *SPS19* amplification occurred considerably earlier compared with *SPS18*. As expected, deletion of *OAF1* in the formerly WT strain resulted in a dramatic 42-fold reduction in *SPS19* expression, validating the phenotype of the mutant generated with the disruption plasmid intended for subsequent integration in the *sum1*Δ strain. Interestingly, this deletant expressed *SPS18* eightfold less efficiently than did the WT. Although the unleashing effect of the *sum1*Δ deletion on *SPS18* expression was not as high in the present experiment compared with the situation in Table 7, nevertheless expression of *SPS18, SMK1*, and *SPS19* was increased. Importantly, deletion of *OAF1* in the *sum1*Δ strain, which was validated by the observation of a sevenfold reduction in *SPS19* expression compared with the WT situation, ended up cancelling the derepressing effect of the *sum1*Δ mutation on *SPS18*.

To confirm these latter results, a further experiment was carried out, in which the cumulative effect of a *pip2*Δ deletion on top of that of *sum1*Δ was examined (Table 9). In this round of oleic acid induction, *SPS19* was almost 1400-fold more highly expressed compared with *SPS18*. In the corresponding *pip2*Δ mutant generated here, *SPS19* was almost tenfold less efficiently expressed compared with the WT, verifying the mutant's phenotype for reduced expression of ORE-regulated genes as a result of the integration of the disruption plasmid. Like the above situation with the *oaf1*Δ deletion, *SPS18* expression in the *pip2*Δ mutant was also affected, albeit to a lesser extent than *SPS19*. Introduction of the *pip2*Δ deletion into the *sum1*Δ mutant resulted in an almost fourfold reduced efficiency in *SPS19* expression by the double deletant compared with the *sum1*Δ deletion alone, thereby authenticating the former's mutant phenotype. The observation made here with *sum1*Δ*pip2*Δ cells, which duplicated that made with the previous *sum1*Δ*oaf1*Δ strain in exposing the overall reduction in *SPS18* expression as a result of altering the oleic acid-induction machinery in the *sum1*Δ mutant, is discussed.

## Discussion

Here, we revealed an important part of the mechanism in *S. cerevisiae* for repressing the sporulation-specific gene *SPS18* under vegetative conditions, and for shielding it from unscheduled transcriptional activation in haploid cells grown on oleic acid. Induction of the divergent partner *SPS19* is instigated by Pip2p-Oaf1p and Adr1p acting at the combined ORE and UAS1 enhancer, a constellation that has been found to activate gene expression synergistically (Gurvitz *et al.*, 2000, 2001; Rottensteiner *et al.*, 2003; Karpichev *et al.*, 2008; Ratushny *et al.*, 2008). The repressing element of *SPS18* was shown here to be comprised of an MSE, but might also involve neighbouring elements including a unidirectionally acting enhancer element UAS^SPS19^ and the M5 element (Fig. 1).

Ndt80p-binding MSEs have the consensus sequence YGNCRCAAA^A^/_T_, and act to upregulate some 300 genes in sporulating diploid cells midway through meiosis (Hepworth *et al.*, 1995, 1998; Ozsarac *et al.*, 1997; Chu *et al.*, 1998). The MSEs in the promoters of the *SMK1, NDT80*, and *SPR3* genes are additionally targeted by Sum1p in association with Hst1p and Rfm1p to repress the corresponding genes in vegetative cells (McCord *et al.*, 2003; Pierce *et al.*, 2003; Xie *et al.*, 1999;). The nucleotide sequence of the MSE in the shared promoter region of *SPS18* and *SPS19* is in very close agreement with the consensus for a Sum1p-binding MSE AGYGWCACAAAAD, with a tolerable G to A deviation at the noncritical position 2 (Pierce *et al.*, 2003). From the findings presented here, the *SPS18* MSE is a repressing element in vegetative cells grown on glucose medium. *SPS18* MSE additionally maintains blockage of *SPS18* transcription under fatty acid-medium conditions, when transcription of *SPS19* is highly active. This was manifested in the situation with the MSE-less version of *SPS18–lacZ* (M3) whose expression was higher in oleic acid-grown WT cells compared with *pip2*Δ*oaf1*Δ mutants, in which the response to oleic acid was impaired, and reinforced by the lowered *SPS18* expression seen in both *sum1*Δ*oaf1*Δ and *sum1*Δ*pip2*Δ deletants as compared with the situation in the parental *sum1*Δ mutant.

Two previous studies place *SPS18* high on the list of oleic acid-induced genes (Koerkamp *et al.*, 2002; Smith *et al.*, 2002;). In one case (Smith *et al.*, 2002), use of diploid cells grown on glycerol before being shifted to oleic acid medium probably introduced an additional physiological response associated with starving cells being synchronized for meiosis and sporulation. This could explain the appearance of *SPS18* in the list of oleic acid-inducible genes, because its expression is a clear indication for onset of sporulation-specific processes (Coe *et al.*, 1994; Chu *et al.*, 1998;). The explanation for why *SPS18* appears on a separate list of highly inducible genes using haploid cells (Koerkamp *et al.*, 2002) rests on the issue of whether the DBY7286 strain used is really WT for expressing *SPS18*. Because DBY7286 is a descendant of S288c (as is the EUROSCARF strain BY4741 used here) that harbours a mutation in the gene for the Hap1p transcription factor, it is modified in many aspects of its respiratory and oxygen metabolism (Gaisne *et al.*, 1999). At least in our hands, real-time amplification of *SPS18* considerably lagged that of *SPS19* in all the experiments.

It is tempting to view the mechanism by which transcriptional regulation of one gene is blocked from affecting that of its diverging gene as a form of insulation. Indeed, insulators in higher eukaryotes are defined in part as elements with the ability to block transcriptional activation of a promoter by a nearby enhancer (Bi & Broach, 1999). The second insulator criterion, to protect transgenes from positive or negative position effects (Bi & Broach, 1999), was not relevant to the present work. However, caution is urged before reclassifying a yeast repressing sequence as an insulator, because at this point it is not clear whether the two mechanisms of action are identical. Nevertheless, it is hoped that the semblance between the end effects orchestrated by these two mechanisms of shielding genes from unscheduled transcription will spur additional studies into this rather underappreciated phenomenon in yeast.

## Statement

Reuse of this article is permitted in accordance with the Creative Commons Deed, Attribution 2.5, which does not permit commercial exploitation.
